# Chromosome Analysis Using Benchtop Flow Analysers and High Speed Cell Sorters

**DOI:** 10.1002/cyto.a.23692

**Published:** 2018-12-17

**Authors:** Bee L. Ng, Beiyuan Fu, Jennifer Graham, Christopher Hall, Sam Thompson

**Affiliations:** ^1^ Cytometry Core Facility Wellcome Sanger Institute Cambridge, CB10 1SA UK; ^2^ Molecular Cytogenetics Facility Wellcome Sanger Institute Cambridge, CB10 1SA UK

**Keywords:** chromosomes, flow karyotype, resolution, bivariate analysis, laser power, flow cytometer, analyser, sorter

## Abstract

The use of the DNA dyes Hoechst (HO) and chromomycin A3 (CA3) has become the preferred combination for the bivariate analysis of chromosomes from both human and animals. This analysis requires a flow cytometer equipped with lasers of specific wavelength and of higher power than is typical on a conventional bench top flow cytometer. In this study, we have investigated the resolution of chromosome peaks in a human cell line with normal flow karyotype using different combinations of DNA dyes on a number of flow cytometers available in a flow cytometry core facility. Chromosomes were prepared from the human cell line using a modified polyamine isolation buffer. The bivariate flow karyotypes of different DNA dyes combination; 4′‐6‐diamidino‐2‐phenylindole (DAPI) or Hoechst with propidium iodide (PI), obtained from different flow cytometers were compared to the reference flow karyotype of DAPI or Hoechst with chromomycin A3, generated from a Mo‐Flo cell sorter using laser power settings of 300 mW each of UV and 457 nm. Good chromosome separation was observed in most of the flow cytometers used in the study. This study demonstrates that chromosome analysis and sorting can also be performed on benchtop flow cytometers equipped with the standard solid state 488 and 355 nm lasers, using a DNA dye combination of DAPI or Hoechst with PI. © 2018 The Authors. Cytometry Part A published by Wiley Periodicals, Inc. on behalf of International Society for Advancement of Cytometry.

## Graphical abstract

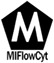

The analysis of chromosome by flow cytometry is termed flow karyotyping. To date, the most complete flow cytometric resolution of the human karyotype is achieved by staining with a combination of DNA dyes namely, Hoechst 33342 (HO) and chromomycin A3 (CA3) 1. The bisbenzimidazole dye HO has a binding preference for adenine‐thymine (AT) rich sequences 2, 3 while the anthraquinones‐based antibiotic CA3 has a binding preference for guanine‐cytosine (GC) rich sequences 4. These DNA stains provide information on the DNA base composition; that is the ratio of AT and GC base pairs, as well as the DNA content of the chromosomes. To obtain high‐resolution flow karyotypes, this dye combination has usually required the use of lasers set in the UV (330–360 nm) and indigo range (440–460 nm) at relatively high‐power settings. While there has been some success in the flow cytometric analysis of chromosomes stained with HO and CA3 using low power air‐cooled lasers 5, 6, the application of flow karyotyping remains limited to laboratories with flow cytometers that are equipped with lasers at these wavelengths. Lasers operating at such wavelength ranges are usually an expensive custom option. Thus, most flow analysers and sorters are equipped with the standard factory optical configuration.

In this study, we investigate the karyotype resolution of a normal human cell line by staining the chromosomes with CA3 and different combinations of DNA dyes specifically DAPI 3 or HO in combination with propidium iodide (PI) and acquired flow karyotypes using standard lasers and optical configuration with flow cytometers available in a flow cytometry core facility.

## Material and Methods

### Cell Culture

Chromosomes were prepared from a normal male human lymphoblastoid cell line GM7016A. The cell line was cultured in RPMI 1640 medium (Thermo Fisher Scientific, UK) supplemented with 10% fetal bovine serum (Thermo Fisher Scientific, UK) and antibiotics (Penicillin and streptomycin; Sigma, UK). The cell line was treated with colcemid (0.1 μg/ml; Sigma, UK) for 5 h after subculturing for 24 h.

### Chromosome Preparation

Chromosomes were prepared by using a polyamine isolation method 7. The blocked cell culture was harvested by centrifuging at 289*g* for 5 min. The cell pel1et was resuspended in 5 ml of hypotonic solution (75 mM KCl, 10 mM MgSO_4_, 0.2 mM spermine, 0.5 mM spermidine, pH 8.0; Sigma, UK) and incubated at room temperature for 30 min.

The suspension of swollen cells was then centrifuged at 289*g* for 5 min. The cell pellet was resuspended in 4 ml of ice cold polyamine isolation buffer (PAB, containing 15 mM Tris, 2 mM EDTA, 0.5 mM EGTA, 80 mM KCl, 3 mM dithiothreitol, 0.25% Triton X‐100, 0.2 mM spermine, 0.5 mM spermidine, pH 7.50; Sigma, UK) and vortexed for about 20–30 s. An aliquot of 5 μl of PAB‐treated cells was mixed with 5 μl of PI to monitor the extent of chromosome separation; chromosomes clumps were dispersed as necessary by further vortexing.

### Chromosome Staining

The chromosome suspension (4 ml) was briefly centrifuged at 201*g* for 2 min and the supernatant was filtered through 20 μm mesh filter (Celltrics, Sysmex, UK) before staining. The filtered chromosome suspension was stained with a combination of DNA dyes. The first dye combination comprised 5 μg/ml of DAPI or Hoechst (Sigma, UK) with 50 μg/ml of chromomycin A3 (Sigma, UK). The second dye combination comprised 5 μg/ml of DAPI or Hoechst (Sigma, UK) with 5 μg/ml of PI (Sigma, UK). The stained chromosome samples were treated with 10 mM MgSO_4_ (Sigma, UK) and 25 mM of sodium sulfite (Sigma, UK) and kept overnight in the dark at 4°C.

### Instrumentation and Optics Configurations

The stained chromosome suspensions were analyzed on a number of flow cytometers available within the core facility. These include benchtop cell analysers; Becton Dickinson (BD) LSRll and LSRFortessa, and high‐speed cell sorters; Mo‐Flo Legacy and BD Influx. The laser power, wavelength, and detection optics were set up specific to each instrument as shown in Table 1.

- BD LSRll
  **Table 1:** Summary of flow cytometer optical configurations, parameters measured and gating strategy

Cytometer	Laser wavelength, Power	Detectors	DNA dyes	Parameters measured	Gating strategy (to exclude clumps and debris)
				(A = Area, H = Height, W = Width)	
Mo‐Flo legacy	MULTILINE UV (330–360 nm), 300 mW	BP 447/60	DAPI, HO	DAPI (H) HO (H)	FSC (H) versus Pulse width
	457 nm, 300 mW	LP 490	CA3	CA3 (H)	
	488 nm, 200 mW	LP 620	PI	PI (H)	
BD Influx	355 nm, 100 mW	BP 460/50	DAPI, HO	DAPI (H) HO (H)	DAPI (H) versus Trigger pulse width
	405 nm, 100 mW	BP 447/60	DAPI, HO	DAPI (H) HO (H)	
	488 nm, 200 mW	LP 620	PI	PI (H)	
	561 nm, 100 mW	BP 610/20	PI	PI (H)	
BD LSRll	355 nm, 20 mW	BP 450/50	DAPI, HO	DAPI (A) HO (A)	DAPI (A) versus DAPI (W) HO (A) versus HO (W)
	405 nm, 25 mW	BP 450/50	DAPI, HO	DAPI (A) HO (A)	
	488 nm, 20 mW	BP 610/20	PI	PI (A)	
BD LSRFortessa	355 nm, 20 mW	BP 450/50	DAPI, HO	DAPI (A) HO (A)	DAPI (A) versus DAPI (W) HO (A) versus HO (W)
	405 nm, 50 mW	BP 450/50	DAPI, HO	DAPI (A) HO (A)	
	488 nm, 50 mW	LP 620	PI	PI (A)	
	561 nm, 50 mW	BP 610/20	PI	PI (A)
  This instrument is a typical 4 laser (488, 405, 355, and 640 nm) cell analyser system with a standard optical set up. In brief, the fluorescence measurement was based on the standard fiber‐optics trigon and octagon detection system. The lasers were all spatially separated at the gel‐coupled flow cell. All the lasers, except 640 nm, were used in this study with 488 nm primarily assigned as the first laser. The laser power for 488 and 355 nm were set at 20 mW and 405 nm was set at 25 mW. The 488 nm laser was used to excite PI and both 405 and 355 nm were used to excite HO and DAPI. Fluorescence was collected using a 610/20 nm band‐pass (BP) filter and a 450/50 nm BP filter for the AT‐specific staining.
- BD LSRFortessa
  Similar to BD LSRll, the BD LSRFortessa is a standard instrument equipped with the optional 561 nm solid‐state laser. The laser power for 488, 405, and 561 nm were all set at 50 mW and 355 nm was set at 20 mW. The lasers 488 and 561 nm were used to excite PI and both 405 and 355 nm were used to excite HO and DAPI. Some optical filter changes were made to the 488 nm light path so that the emission was collected on the first detector of the octagon detection pod (marked as “A” as shown in diagram on supplier website) (https://www.bdbiosciences.com/sg/instruments/facscantoresearch/features/optics.jsp). Fluorescence emitted from PI excited by 488 nm was collected using a 620 m long pass (LP) filter and with a 610/20 nm BP filter from 561 nm excitation. Fluorescence emitted from HO and DAPI was collected using a 450/50 nm BP filter.
- BD INFLUX
  This instrument has been modified with the fluorescence measurement made on a fiber‐optics based trigon and octagon detection system similar to the BDLSR series analyser. This stream‐in‐air sorter is equipped with five solid‐state lasers but only four of the lasers were used in this study. The laser power for 561, 405, and 355 nm were all set at 100 mW and 488 nm was set at 200 mW. The 488 and 561 nm lasers were used to excite PI and both 405 and 355 nm were used to excite HO and DAPI. Some optical filter changes were made to the 488 and 405 nm light paths so that the emission was collected on the first detector of the octagon detection pod (marked as “A” as shown in diagram on supplier website) (https://www.bdbiosciences.com/sg/instruments/facscantoresearch/features/optics.jsp). Fluorescence emitted from PI excited by 488 nm was collected using a 620 nm LP filter and with a 610/20 BP filter from 561 nm excitation. Fluorescence emitted from HO and DAPI excited by 355 nm was collected using a 460/50 nm BP filter and with a 447/60 BP filter from 405 nm excitation.
- Mo‐Flo Legacy
  This instrument has a Z‐configuration optics platform and is equipped with two water‐cooled lasers (Coherent, Innova 300 series) and a 488 nm solid‐state laser spatially separated at the stream‐in‐air flow chamber. The primary water‐cooled laser was tuned to emit light at 457 nm (indigo) to excite CA3 and the secondary laser was tuned to multiline UV (330–360 nm) to excite Hoechst or DAPI. The power of both water‐cooled lasers was set at 300 mW and kept constant using light control feedback. The solid‐state 488 nm laser shared the first pin‐hole with the 457 nm laser and was set at 200 mW for PI excitation. Fluorescence emitted from CA3 was collected using a 490 nm LP filter, PI using a 620 nm LP filter, and HO and DAPI using a 447/60 nm BP filter.

### Flow Cytometric Analysis

The performance of the bench‐top analysers was checked using CST beads (BD™ Cytometer Set up and Tracking Beads, BD Biosciences) and Quality Control (QC) passed before chromosome analysis. For the cell sorters, the optical light path was aligned before chromosome analysis using 3 μm beads (Sphero™ Rainbow Fluorescent particles, Spherotech) obtaining a minimum peak coefficient of variance for all fluorescence channels. The instrumentation and optical configuration required for each fluorochrome used on each respective instrument, the parameters measured and its respective gating strategy used to remove clumps and debris are summarized in Table 1.

The stained chromosome suspensions were analyzed on the flow cytometers and a total of 50,000 events acquired and displayed on a bivariate plot of Hoechst (HO) or DAPI versus PI after gating out clumps and debris on HO or DAPI fluorescence versus respective pulse width except for the analysis made on the Mo‐Flo and BD Influx which was gated on FSC versus pulse width 8 and DAPI fluorescence versus pulse width, respectively (Table 1). The data obtained from these dye combinations were compared to the chromosome plot of HO or DAPI versus CA3 and HO or DAPI versus PI collected from Mo‐Flo. For the calculation of median fluorescence, ellipsoid regions were placed around the chromosomes of interest on the bivariate flow karyogram and the median of the gated regions were calculated univariately for DAPI, HO, and PI fluorescence using Flowjo v10 (Flowjo LLC.). Chromosomes identified on the plots were numerically labeled with reference to the ISCN (International system for Human Cytogenetic Nomenclature) standard. Unexpected chromosome peaks discovered on the karyotype plot were sorted and verified by chromosome painting as described previously 9, 10.

## Results

### Flow Karyotype of GM7016A Using the Dye Combination DAPI and PI

**Figure 1:**
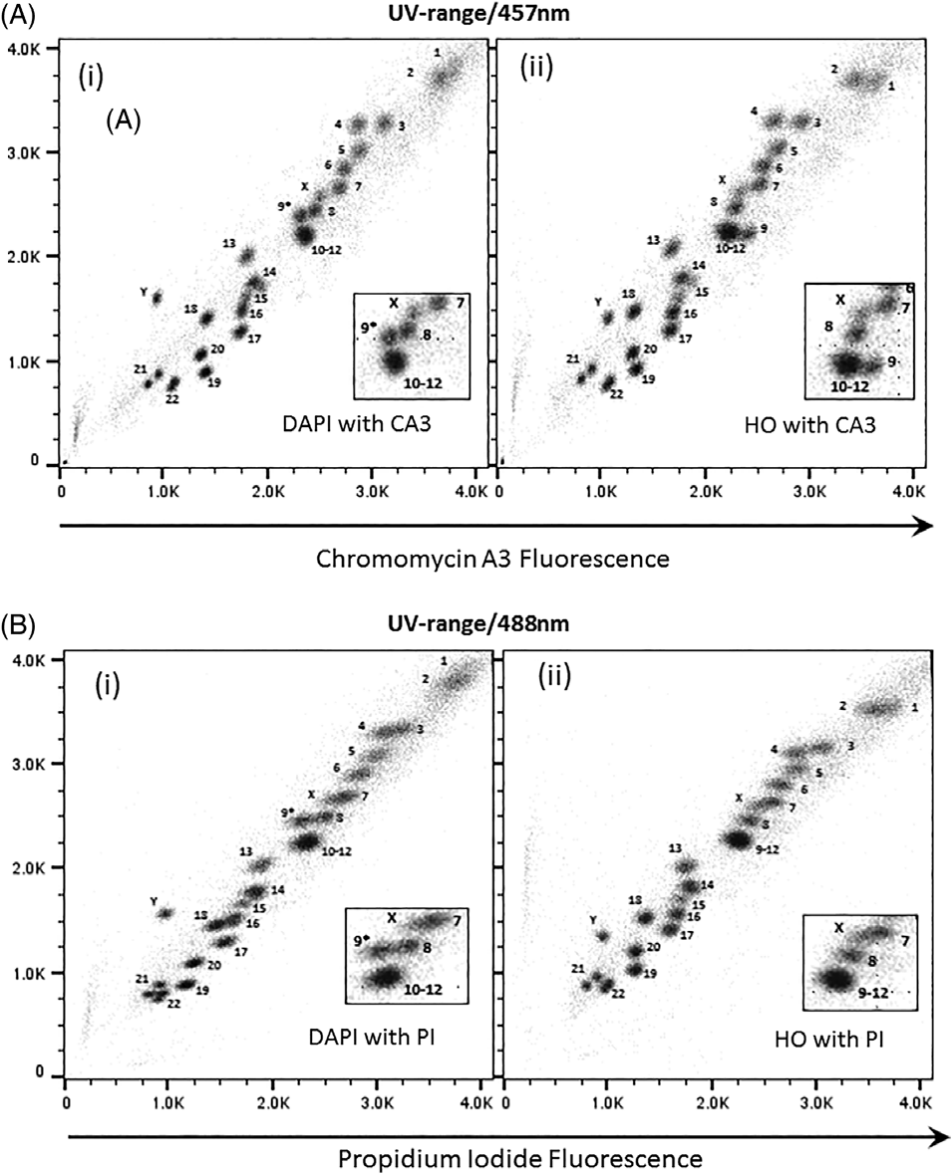
Bivariate flow karyotypes plot of chromosomes from a normal male human lymphoblastoid cell line, GM7016A. Chromosomes were stained with different dye combinations acquired from sorter MOFLO Legacy with AT‐specific stain fluorescence on the *y*‐axis. (**A**) Lasers settings of 300 mW UV to excite DAPI (i) or HO (ii) and 457 nm to excite CA3. (**B**) Lasers settings of 300 mW UV to excite DAPI (i) or HO (ii) and 488 nm at 200 mW to excite PI. The inset panel shows the 9–12 cluster in more detail. The unexpected chromosome peak is indicated (9*).

**Figure 2:**
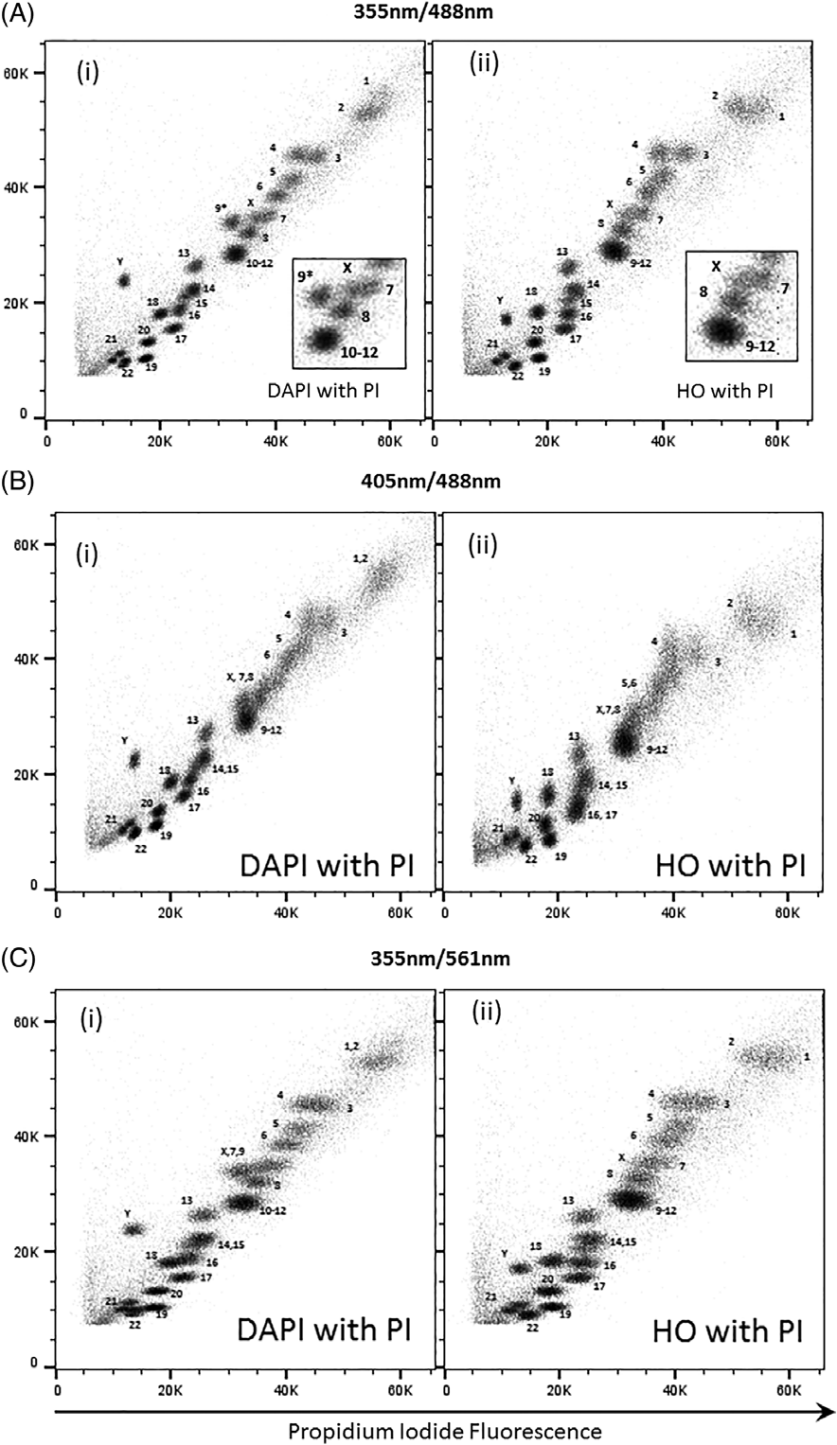
Bivariate flow karyotypes plot of chromosomes from a normal male human lymphoblastoid cell line, GM7016A. Chromosomes were stained with different dye combinations acquired from sorter BD INFLUX with AT‐specific stain fluorescence on the *y*‐axis. (**A**) Lasers settings of 100 mW 355 nm to excite DAPI (i) or HO (ii) and 488 nm at 200 mW to excite PI. (**B**) Lasers settings of 100 mW 405 nm to excite DAPI (i) or HO (ii) and 488 nm at 200 mW to excite PI. (**C**) Lasers settings of 100 mW 355 nm to excite DAPI (i) or HO (ii) and 561 nm at 100 mW to excite PI. The inset panel shows the 9–12 cluster in more detail. The unexpected chromosome peak is indicated (9*).

**Figure 3:**
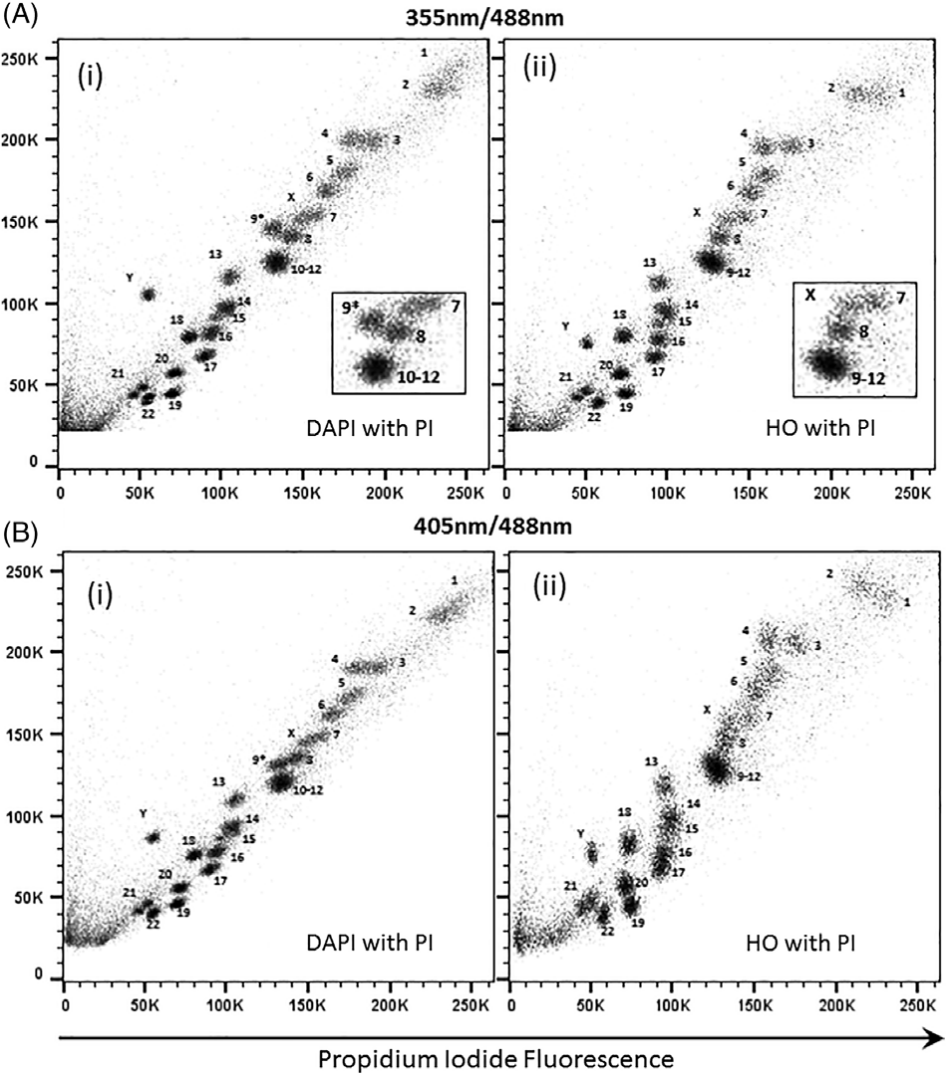
Bivariate flow karyotypes plot of chromosomes from a normal male human lymphoblastoid cell line, GM7016A. Chromosomes were stained with different dye combinations acquired from analyser BD LSRll with AT‐specific stain fluorescence on the *y*‐axis. (**A**) Lasers settings of 20 mW 355 nm to excite DAPI (i) or HO (ii) and 488 nm at 20 mW to excite PI. (**B**) Lasers settings of 25 mW 405 nm to excite DAPI (i) or HO (ii) and 488 nm at 20 mW to excite PI. The inset panel shows the 9–12 cluster in more detail. The unexpected chromosome peak is indicated (9*).

**Figure 4:**
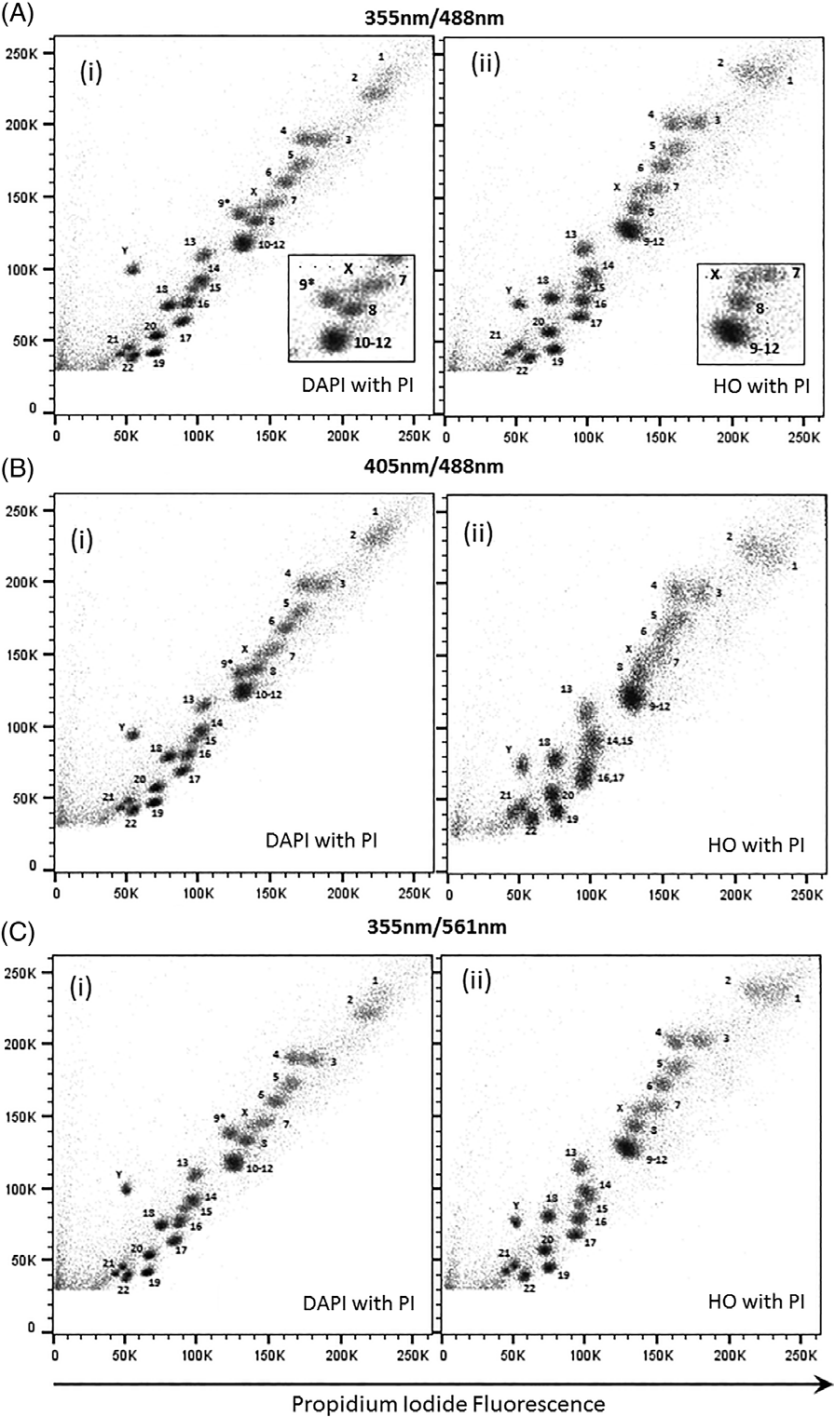
Bivariate flow karyotypes plot of chromosomes from a normal male human lymphoblastoid cell line, GM7016A. Chromosomes were stained with different dye combinations acquired from analyser BD LSRFortessa with AT‐specific stain fluorescence on the *y*‐axis. (**A**) Lasers settings of 20 mW 355 nm to excite DAPI (i) or HO (ii) and 488 nm at 50 mW to excite PI. (**B**) Lasers settings of 50 mW 405 nm to excite DAPI (i) or HO (ii) and 488 nm at 50 mW to excite PI. (**C**) Lasers settings of 20 mW 355 nm to excite DAPI (i) or HO (ii) and 561 nm at 50 mW to excite PI. The inset panel shows the 9–12 cluster in more detail. The unexpected chromosome peak is indicated (9*).

The flow karyotype of the human lymphoblastoid cell line GM7016A labeled with DAPI and PI and measured using UV and 488 nm lasers is shown in Figures 1B(i), 2A(i), 3A(i), and 4A(i). The resolution of the flow karyotype obtained from all the flow cytometers was comparable. With this dye combination and laser settings, a total of 24 chromosome clusters was revealed with 21 of them distinctively resolved from their neighbors with the exception of the chromosome 10–12 cluster. A new chromosome peak, not observed in the standard HO and CA3 dye combination, was noted located near to the chromosome 8 peak (Fig. 1B(i), inset). This was flow sorted and verified to be normal chromosome 9 by chromosome painting (labeled as 9* on karyotype plot). Similarly, this chromosome 9* peak was also observed with the DAPI and CA3 dye combination (Fig. 1A(i), inset) on the karyotype plot obtained from the Mo‐Flo. The chromosome 10–12 cluster was also isolated and confirmed to contain only three pairs of chromosomes (i.e., chromosome 10–12, data not shown) by chromosome painting. In addition, the chromosome Y peak was observed to be further separated from the rest of the chromosomes with DAPI dye combination (Figs. 1A–B(i), 2A–C(i), 3A–B(i), and 4A–C(i)) compared to the standard flow‐karyotype. The fluorescence intensity for the chromosome Y peak compared to chromosome 18 was observed to be greater with DAPI (average 1.26‐fold) but less with HO (average 0.93‐fold). There was no significance difference in the fluorescence intensity of PI for both dye combinations (Table 2). In the standard karyotype using HO with CA3, the larger sized chromosomes 1 and 2 displayed next to one another in horizontal orientation. With DAPI, the chromosome 1 peak was displaced vertically above chromosome 2 (Figs. 1A–B(i), 2A–C(i), 3A–B(i), and 4A–C(i)).

**Table 2:** HO, DAPI, and PI median fluorescence of gated events of chromosome 18 and Y from a normal male human lymphoblastoid cell line, GM7016A measured using UV and 488 nm lasers

	DAPI/PI						HO/PI					
Dye combinations	DAPI median fluorescence			PI median fluorescence			HO median fluorescence			PI median fluorescence		
Flow cytometer	18	Y	Ratio (Y/18)	18	Y	Ratio (Y/18)	18	Y	Ratio (Y/18)	18	Y	Ratio (Y/18)
Mo‐Flo	1,458	1,566	1.07	1,455	967	0.66	1,526	1,349	0.88	1,356	951	0.7
BD Influx	18,176	23,824	1.31	19,968	13,600	0.68	18,464	17,184	0.93	18,176	12,784	0.7
BD LSRll	79,552	105,216	1.32	80,000	54,976	0.69	80,000	75,520	0.94	72,832	50,496	0.69
BD LSRFortessa	74,752	99,136	1.33	79,424	54,336	0.68	80,960	76,800	0.95	74,688	52,032	0.70
Average			1.26			0.68			0.93			0.70

### Flow Karyotype of GM7016A Using Dye Combination HO and PI

The flow karyotype of a human lymphoblastoid cell line GM7016A stained with HO and PI measured using UV and 488 nm lasers is shown in Figures 1B(ii), 2A(ii), 3A(ii), and 4A(ii). The resolution of the flow karyotypes obtained from all the flow cytometers using this dye combination was comparable. A total of 24 chromosome clusters was observed with 21 of them distinctively resolved from their neighbors. The chromosome Y peak was resolved from the rest of the chromosomes but with less separation when compare to staining with DAPI. The orientation of chromosomes 1 and 2 was comparable to the standard HO and CA3 staining (Fig. 1A(ii)).

### Flow Karyotype of GM7016A Using a 405 nm Laser to Excite AT‐Specific Stain (DAPI, HO) and a 488 nm Laser to Excite PI

The chromosome samples were analyzed using lasers at 488 nm to excite PI and 405 nm to excite the AT‐specific stains DAPI and HO. The resolution of the flow karyotypes obtained from the DAPI and PI dye combination for both analysers BD LSRll and BD LSR Fortessa was comparable (Figs. 3B(i) and 4B(i)) to the karyotype obtained from Mo‐Flo at high power UV‐range laser setting (Fig. 1B(i), 300 mW). For the HO and PI dye combination, a loss in the data resolution was observed from both analysers (Figs. 3B(ii) and 4B(ii)) as well as from the stream‐in‐air BD Influx cell sorter (Fig. 2B(ii)) whereby a significant number of chromosome peaks were unresolved.

### Flow Karyotype of GM7016A Using 355 nm Laser to Excite AT‐Specific Stain (DAPI, HO) and 561 nm Laser to Excite PI

The chromosome samples were analyzed using 355 nm to excite the AT‐specific stains DAPI and HO and a 561 nm to excite PI. In this experiment, only BD LSR Fortessa and BD Influx were used, as both instruments were supplied with a 561 nm. The resolution of the chromosome peaks with 561 nm excitation (Fig. 4C) was comparable to the karyotype obtained from Mo‐Flo high power using the 488 nm laser configuration (Fig. 1B).

## Discussion and Conclusions

High‐powered water‐cooled lasers have generally been required to produce the excitation needed for the generation of well‐resolved chromosome data by flow cytometry 9. Here, we investigate the possibility of flow karyotyping using different dye combinations on flow cytometers with standard optical configuration (Table 1).

We have found that all the analysers used in this study were able to resolve most of the chromosome peaks using the DAPI or HO with PI dye combination using UV and 488 nm laser settings (Figs. 3A(i)–(ii) and 4A(i)–(ii)). The data resolution acquired using this dye combination was comparable to that obtained from Mo‐Flo at high‐powered laser settings (Fig. 1B). All the lasers used in the study, except the UV and 457 nm laser from Mo‐Flo, were all solid‐state lasers. To achieve this degree of data resolution, much higher laser power is required for the stream‐in‐air sorters such as the BD Influx and Mo‐Flo. Both analysers made used of “cuvettes quartz type” flow chambers 11 which are gel‐coupled to the collection optics. We suggest that it is this optical feature that enhances the light collection emitted from the stained chromosomes allowing the acquisition of well‐resolved chromosome peaks even at low laser power settings.

Interestingly, a new chromosome peak was discovered in the DAPI and PI or DAPI and CA3 dye combination (Fig. 1A(i),B(i)). This was verified to be chromosome 9 by chromosome painting. We have noted that this heteromorphic chromosome 9 peak, which is positioned near to the 9–12 chromosome cluster in this cell line in the standard HO and CA3 karyotype plot (Fig. 1A(ii)), was transposed next to the chromosome 8 peak in the DAPI dye combination. We have also found that the peaks of other chromosomes, such as chromosome 1 and Y, were separated further on the “AT‐rich” axis with all the flow cytometers used in the study. This observation has also been reported previously with a 4′‐6‐bis[2′‐imidazolinyl‐4H,5H]‐2‐phenyl‐indole (DIPI) dye combination 12. The polymorphic nature of these chromosomes, particularly involving AT‐rich heterochromatin, influences the binding properties of the dye combination used thus staining the chromosomes more brightly and displacing the chromosome peak to a “new AT‐rich” position 13, 14.

Conventionally, bivariate analysis of chromosomes requires a pair of base‐specific fluorescent dyes to resolve chromosome types based on their difference in base composition and DNA content. Previous studies have shown that PI on its own has no binding preference for base‐specific sequences 15, 16. In this study we show that PI, when used in combination with DAPI or HO dyes, can resolve most of the chromosomes peaks display in the flow karyotype using the chromosome staining conditions detailed in this study. In fact, a well‐resolved chromosome profile comparable to that of Mo‐Flo was achievable from the analysers at low laser power (20 mW of 355 nm, 20–50 mW of 488 nm) using DAPI or HO in combination with PI. Similar chromosome resolution was also acquired from the sorter BD Influx using this dye combination albeit at a much higher laser powers (100 mW of 355 nm, 200 m of 488 nm). We speculate that this binding phenomenon could either be due to the spectral energy transfer or binding competition which occurs between the pair of DNA dyes used 17, 18. However, the chemistry behind the chromosomal binding properties of PI with other DNA dyes is beyond the scope of this article.

We have also made use of the yellow green 561 nm laser available on one of the analysers and a sorter for the excitation of PI. However, no significance improvement in the chromosome data resolution was observed despite the fact that PI has almost twice the excitation efficiency at 561 nm (71%) compared to 488 nm (37%) (data from BioLegend Spectra Analyser tool, https://www.biolegend.com/spectraanalyzer). Surprisingly, on the analysers, we were able to obtain comparably highly resolved chromosome data using 405 nm laser excitation for the DAPI dye combination although the efficiency of dye excitation was only 7% at this wavelength. However, a loss of chromosome resolution was observed using the 405 nm excitation line on the stream‐in‐air BD Influx sorter with the HO and PI dye combination.

Here, for the first time, we have demonstrated that flow karyotypes with well‐resolved chromosome peaks can be acquired using a combination of either DAPI or HO with PI on flow cytometers equipped with typical lasers and optical configuration. The technique for chromosome preparation 7 is simple to follow and the chromosome analysis can be easily carried out in any laboratories who own a flow cytometer with a blue 488 nm and UV 355 nm laser line. This staining technique, together with the advance of lower cost sequencing, will extend the application of flow karyotyping in chromosome genomics for both human and animals.

## Supporting information


**Appendix S1:** MIFlowCyt Item Checklist‐Ng
