# Supplementary material for: Chromosome Analysis Using Benchtop Flow Analysers and High Speed Cell Sorters
Source: Cytometry A. 2018 Dec 17;95(3):323–31. doi: 10.1002/cyto.a.23692 (PMC6491968; doi:10.1002/cyto.a.23692)
Supplement: Supplementary file 1 — Appendix S1: MIFlowCyt Item Checklist‐Ng [file CYTO-95-323-s001.docx]

**Cytometry Part A**

**Author Checklist: MIFlowCyt-Compliant Items**

| **Requirement** | **Please Include Requested Information** |
| --- | --- |
| 1.1. Purpose | Chromosome analysis on benchtop flow cytometer |
| 1.2. Keywords | Chromosomes; flow karyotype; resolution; bivariate analysis, laser power; flow cytometer; analyser; sorter |
| 1.3. Experiment variables | Data resolution, Fluorescence median intensity HO, DAPI, PI |
| 1.4. Organization name and address | Cytometry Core Facility, Wellcome Sanger Institute, Hinxton, Cambridge, CB10 1SA, UK |
| 1.5. Primary contact name and email address | Bee Ling Ng. Email: bln@sanger.ac.uk |
| 1.6. Date or time period of experiment | Sept2017 –Aug2018 |
| 1.7. Conclusions | We were able to perform flow karyotyping on benchtop flow cytometers. |
| 1.8. Quality control measures | CST QC beads from Becton Dickinson and 3µm Rainbow beads from Spherotech for laser alignment. |
| 2.1.1.1. (2.1.2.1., 2.1.3.1.) Sample description | Human cell line |
| 2.1.1.2. Biological sample source description | Chromosome from a normal human lymphoblastoid cell line, GM7016A |
| 2.1.1.3. Biological sample source organism description | Human |
| 2.1.2.2. Environmental sample location | NA |
| 2.3. Sample treatment description | Cell line treated with colcemid for 5hr. |
| 2.4. Fluorescence reagent(s) description | DAPI, Hoechst, Chromomycin A3, Propidium Iodide |
| 3.1. Instrument manufacturer | Becton Dickinson, Beckman Coulter (service by Propel Labs) |
| 3.2. Instrument model | BDLSRll, BDLSRFortessa, Mo-Flo Legacy, BDInflux |
| 3.3. Instrument configuration and settings | Please refer to ‘Instrumentation and optics configuration’ section and Table 1 in the manuscript |
| 4.1. List-mode data files | *We recommend all authors to submit their data files to [http://flowrepository.org](http://flowrepository.org/) and to make them available for the peer-review process. If you have done so, please let us know by inserting the following codes (replace the red text):  1) The link for peer-review process:  https://flowrepository.org/id/RvFrBO1Z2xkeuKPh0Vm40ylitYsPybSMGp3GMy2oMgVVqlJ2xwv6iDZ5crxQTqMU  This link will only be shared with reviewers of your manuscript.  2) The repository identifier:  http://flowrepository.org/id/FR-FCM-ZYX6. This link will be made publicly accessible after the paper is published. |
| 4.2. Compensation description | None |
| 4.3. Data transformation details | None |
| 4.4.1. Gate description | On a bivariate plot of Hoechst (HO) or DAPI (DA) versus Propidium Iodide (PI) after gating out clumps and debris on HO or DA fluorescence versus respective pulse width except for the analysis made on the Mo-Flo and BD Influx which was gated on FSC versus pulse width and DAPI fluorescence versus pulse width respectively.  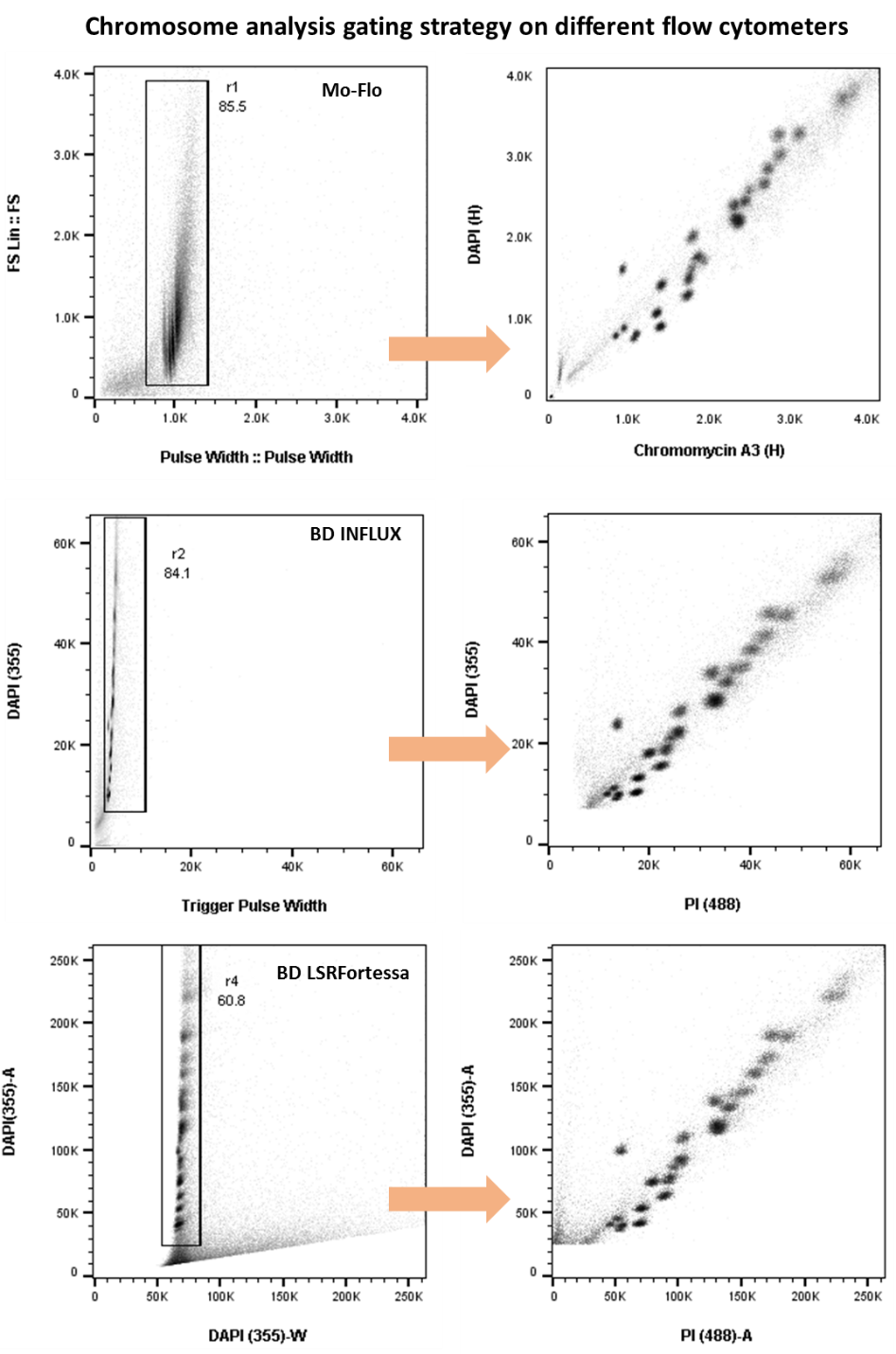 |
| 4.4.2. Gate statistics | Please refer to Table 2 in the manuscript |
| 4.4.3. Gate boundaries | None |

**Notes**

Feel free to use more space than allocated.

You can embed graphics/figures in this document, if needed.

Please make sure to save the document in Microsoft Word version 2003 or older, before uploading to ScholarOne Manuscripts. When uploading this checklist to ScholarOne Manuscripts, please choose the “Supplementary Material for Review” category.

Please note that if your paper is accepted, the checklist will be published as an Online Supporting Information.

For any questions, please contact the Cytometry Part A editorial office at [Cytometrya@wiley.com](mailto:Cytometrya@wiley.com).
